# Systematic Analyses of the Cytotoxic Effects of Compound 11a, a Putative Synthetic Agonist of Photoreceptor-Specific Nuclear Receptor (PNR), in Cancer Cell Lines

**DOI:** 10.1371/journal.pone.0075198

**Published:** 2013-09-16

**Authors:** Zibo Zhao, Lu Wang, Zhi Wen, Serife Ayaz-guner, Yidan Wang, Paul Ahlquist, Wei Xu

**Affiliations:** 1 McArdle Laboratory for Cancer Research, University of Wisconsin-Madison, Madison, Wisconsin, United States of America; 2 Institute for Molecular Virology, University of Wisconsin-Madison, Madison, Wisconsin, United States of America; 3 Howard Hughes Medical Institute, University of Wisconsin-Madison, Madison, Wisconsin, United States of America; 4 Morgridge Institute for Research, University of Wisconsin-Madison, Madison, Wisconsin, United States of America; 5 Department of Oncology, University of Wisconsin-Madison, Madison, Wisconsin, United States of America; Van Andel Research Institute, United States of America

## Abstract

Photoreceptor cell-specific receptor (PNR/NR2E3) is an orphan nuclear receptor that plays a critical role in retinal development and photoreceptor maintenance. The disease-causing mutations in PNR have a pleiotropic effect resulting in varying retinal diseases. Recently, PNR has been implicated in control of cellular functions in cancer cells. PNR was reported to be a novel regulator of ERα expression in breast cancer cells, and high PNR expression correlates with favorable response to tamoxifen treatment. Moreover, PNR was shown to increase p53 stability in HeLa cells, implying that PNR may be a therapeutic target in this and other cancers that retain a wild type p53 gene. To facilitate further understanding of PNR functions in cancer, we characterized compound 11a, a synthetic, putative PNR agonist in several cell-based assays. Interestingly, we showed that 11a failed to activate PNR and its cytotoxicity was independent of PNR expression, excluding PNR as a mediator for 11a cytotoxicity. Systematic analyses of the cytotoxic effects of 11a in NCI-60 cell lines revealed a strong positive correlation of cytotoxicity with p53 status, i.e., p53 wild type cell lines were significantly more sensitive to 11a than p53 mutated or null cell lines. Furthermore, using HCT116 p53+/+ and p53-/- isogenic cell lines we revealed that the mechanism of 11a-induced cytotoxicity occurred through G_1_/S phase cell cycle arrest rather than apoptosis. In conclusion, we observed a correlation of 11a sensitivity with p53 status but not with PNR expression, suggesting that tumors expressing wild type p53 might be responsive to this compound.

## Introduction

Nuclear hormone receptors regulate a variety of essential biological processes including development, differentiation and cell survival [1-3]. Their activities and expression levels are tightly controlled, and dysregulation of nuclear receptors (NRs) and their coregulators is involved in metabolic diseases and cancer development [4-6]. NRs are the second largest family of proteins that are targeted by pharmaceutical drugs [7]. Of the 48 nuclear receptors identified in humans, approximately half are well-characterized with known natural ligands. The remaining NRs are so called orphan nuclear receptors because their physiological ligands remain unknown. Despite having no natural ligands, orphan nuclear receptors can be targeted with synthetic ligands for treatment of human diseases, e.g. synthetic ROR and LRH-1 agonists were used to treat metabolic and autoimmune diseases [8]. Fluorescent polarization assays, amplified luminescent proximity homogeneous (ALPHAScreen) assays, and time-resolved fluorescence energy transfer (TR-FERT) assays have been developed as high throughput screening (HTS) approaches to identify compounds that target nuclear receptors for therapeutic purposes [9-12].

NR2E3/PNR is an orphan nuclear receptor that is highly expressed in retinal cells [13] and modestly expressed in prostate and uterine tissues [14,15]. PNR activates rod-specific gene expression and suppresses cone-specific gene expression by down-regulating cyclin D1 and TBX2 [16-20]. This gene regulation pattern defines the dual role of PNR in mediating the development and maintenance of photoreceptors [21]. Mutations in PNR have been found in various retinal diseases, including enhanced S-cone syndrome, autosomal dominant and recessive forms of retinitis pigmentosa, Goldmann-Favre syndrome, and clumped pigmentary retinal degeneration [22-27]. Emerging evidence suggests that PNR might have important functions in cancer cells by regulating p53 stability and estrogen receptor alpha (ERα) expression. In HeLa and HCT116 p53-positive cancer cell lines, PNR stabilizes p53 by acetylation and induces apoptosis [28]. In the ERα-positive breast cancer cell lines MCF7 and T47D, PNR regulates ERα by directly binding to the ERα promoter region, thereby increasing ERα gene expression [29]. The expression of PNR is also significantly associated with recurrence-free survival and favorable tamoxifen response in ERα-positive, node negative breast cancer patients [29]. These studies imply that PNR might be a therapeutic target for retinal diseases, cancers retaining a wild type p53 gene, and ERα-positive breast cancers.

PNR specific agonists, either natural or synthetic, have been identified using high throughput screening assays. Because apo-PNR has been shown to interact with co-repressors N-COR, SMRT, and RetCoR [20,30], the synthetic PNR agonist compound 11a was identified using a GAL4 DNA binding domain-PNR ligand binding domain fusion β-lactamase transactivation assay and NCOR release assay [30,31]. Although 11a was tested in cell-based assays for agonistic effects on PNR and was shown to have low toxicity in control cell lines, 11a has not been shown to bind PNR directly. Rather, recent evidence suggests that 11a is unlikely to be a direct PNR agonist [32]. Our result agrees with this later conclusion. As PNR was recently implicated in ERα positive breast cancer and shown to regulate p53 stability, this compound may have therapeutic utility. However, systematic evaluation of compound cytotoxicity was lacking and the cellular targets of 11a have not yet been defined. In this study, we systematically evaluated the cytotoxic effects of 11a in NCI-60 cell lines [33] and found that 11a cytotoxicity is independent of PNR expression but positively correlates with p53 status, with higher sensitivity in p53 wild type cell lines than p53 null/mutant cell lines. Using HCT116 p53+/+ and p53-/- isogenic cell lines, we demonstrated that the cytotoxic effects of 11a largely resulted from p53-induced G_1_/S phase cell cycle arrest, with minor contribution from apoptosis.

## Materials and Methods

### Cell culture and 11a treatment

The LM2 cell line was a kind gift from Dr. Joan Massagué [34]. The HCT116 isogenic cell lines were a kind gift from Dr. B. Vogelstein [35]. All of the other cell lines were purchased from the American Type Culture Collection (Rockville, MD). The HEK293T, MCF7, MDA-MB-231, LM2, MDA-MB-468, SKOV3, and HCT116 isogenic cell lines were maintained in Dulbecco’s modified Eagle’s medium (DMEM) (Gibco, Gaithersburg, MD) supplemented with 10% fetal bovine serum (FBS) (Gibco) at 37°C with 5% CO_2_. The A2780 and OVCAR3 ovarian cancer cell lines were maintained in RPMI-1640 (Gibco) supplemented with 10% FBS. The T47D breast cancer cell line was maintained in DMEM/F12 (Gibco) supplemented with 10% FBS. Compound 11a was purchased from Pharmabridge Inc. (Pennsylvania Biotechnology Center, Doylestown, PA). The 11a powder was dissolved in ethanol first and then in dimethyl sulfoxide (DMSO) (Sigma Chemical Co., St. Louis, MO) at a final concentration of 8 mM. Asynchronous cells were seeded 24 hours before treatment with 11a, such that cells were approximately 50%-60% confluent at the time of 11a addition. The final concentration of 11a in nM - μM range was achieved by diluting 11a in the fresh medium, and 0.1% DMSO was used as the control for each experiment. All-trans retinoic acid, doxorubicin, etoposide, staurosporine and 3-aminobenzamide were purchased from Sigma (St. Louis, MO).

For cell cycle analysis, cells were serum-starved for 24 hours in order to achieve G_0_ synchronization. The cells were then allowed to re-enter the cell cycle by supplementing with DMEM plus 10% FBS containing the indicated concentrations of 11a.

### Retrovirus packaging, infection and stable cell line generation

The packaging plasmids pME-VSVG, pHIT60 and pLNCX were purchased from OpenBiosystems (Huntsville, AL). Retroviruses were packaged in HEK293T cells transfected with 3.8 µg pME-VSVG, 1.4 µg pHIT60 and 3.8 µg pLNCX-GFP or pLNCX-PNR using transIT-LT1 reagent (Mirus Bio) according to the manufacturer’s instructions. Six hours after transfection, the medium was changed. The virus particles were then harvested 24 to 48 hours later using a 0.45 µm syringe filter (Thermo Scientific).

To infect the cells with retroviruses, the viruses were mixed with an equal volume of fresh media supplemented with 10% FBS. Polybrene was added at a final concentration of 5 µg/mL in order to increase the infection efficiency. The medium was changed 6 hours after infection. Cells were selected with G418 (800 µg/ml) for a week to generate stable cell lines expressing GFP or PNR.

### CellTiter Glo luminescent cell viability assays

One thousand cells per well were seeded in quadruplicate in a 384-well plate and treated with the indicated concentrations of 11a for a week. Cells were then subjected to the CellTiter Glo luminescent cell viability assay according to the manufacturer’s instructions (Promega, Madison, WI). The IC_50_ values were calculated by using the XLfit^TM^ add-in for Excel.

### Luciferase reporter assays

The DR2-driven luciferase reporter, TLX and COUP-TFI plasmids were kind gifts from Dr. Ronald Evans. COUP-TFII plasmid was a kind gift from Dr. Michael Gould. The other plasmids were purchased from OpenBiosystems (Huntsville, AL). Luciferase assays were performed using the Luciferase Assay System (Promega, Madison, WI). HEK293T cells were seeded in a 96-well plate (2 × 10^3^/well). After 24 hours, cells were transfected using transIT-LT1 (Mirus Bio) with 20ng DR2-driven luciferase reporter, 10 ng β-galactosidase reporter, and 20ng CMV expression vector for control, PNR, TLX, COUP-TFI or COUP-TFII. Compound 11a was added 24 hours after transfection, and luciferase activity was determined after incubation for an additional 24 hours. β-galactosidase activity was used to normalize for transfection efficiency.

### Cell proliferation assays

Cells (2 × 10^3^/well) were seeded on a 96-well plate. After 24 hours, various concentrations of 11a were added to the plates. The cells were cultured for 72 hours and then 3-(4,5-dimethylthiazol-2-yl)-2,5-diphenyltetrazolium (Sigma-Aldrich) solution (20 µl per well, 4mg/ml in PBS) was added. The cells were incubated at 37°C for 4 hours. After discarding the supernatant, 200 µl DMSO was added and the absorbance was measured with a 540 nm filter on a Victor X5 microplate reader (PerkinElmer, Waltham, MA). Approximate IC_50_ values were calculated using GraphPad Prism Software (Version 5.04, Graph-Pad Software Inc., San Diego, CA) and a three parameter log versus response nonlinear regression.

### Cell Cycle Analysis

Cells were harvested by trypsinization, centrifuged and fixed in 80% ice-cold ethanol dropwise with continuous vortexing. Before analysis, cells were centrifuged, and the ethanol was removed. The cell pellets were resuspended in 1 ml PI/RNase solution (50 µg/ml propidium iodide, 50 µg/ml RNase A, 0.25% Triton X-100 in PBS). The flow cytometry analysis was performed with a FACSCalibur (BD Biosciences, San Jose, CA) with excitation at 488 nm. Integrated red fluorescent histograms were analyzed with Modfit LT (Verity House Software, Topsham, ME).

### Apoptosis assay measured by Annexin V/PI staining

Cells were stained with Alexa-488 Annexin V and PI, and evaluated for apoptosis by flow cytometry according to the manufacturer’s protocol (Invitrogen). Briefly, 1×10^6^ cells were washed twice with PBS, and stained with 5 µl of Annexin V and 1 µl of PI (100 µg/ml) in 1× binding buffer for 15 min at room temperature in the dark. The flow cytometry analysis was performed with the FACSCalibur. Both early apoptotic (annexin V-positive, PI-negative) and late (annexin V-positive and PI-positive) apoptotic cells were included in cell death determinations analyzed by FlowJo (Tree Star Inc., Ashland, OR).

### Western Blot Analysis

Cells were harvested and lysed with RIPA buffer (50mM Tris, 150mM NaCl, 0.1% SDS, 0.5% sodium deoxycholate, 1% Triton X 100, 1mM DTT, protease inhibitors and benzonase). After centrifugation, total protein was quantified using the BioRad Protein Assay (BioRad), and 25 µg of protein was resolved SDS-PAGE. Proteins were transferred to a nitrocellulose membrane for 1.5 hours at 0.35 A. Membranes were blocked with 5% nonfat milk and incubated with primary antibody at room temperature for 2 hours or overnight. Membranes were then incubated with secondary antibody for 1 hour at room temperature and visualized using SuperSignal West Pico Chemiluminescent Substrate (Thermo Scientific, Waltham, MA) on autoradiography film. Anti-PNR antibody was generated by the Genemed Synthesis Inc., TX. Two KLH-conjugated peptides were synthesized by Genemed Synthesis Inc. : PETRGLKDPEHVEALQD and LSQHSKAHHPSQP, corresponding to human PNR amino acids 331-347 and 353-365, respectively. These peptides were used to immunize rabbits. The antiserum was affinity purified after the final bleed to obtain anti-PNR specific antibody. Anti-p53 and anti-p21 antibodies were obtained from Pierce (Rockford, IL); anti-Cyclin D1 and anti-Hsp90 antibodies were obtained from Santa Cruz Biotechnology (Santa Cruz, CA); anti-PARP antibody was obtained from Cell Signaling Technology (Danvers, MA).

### Quantitative Real-Time PCR analysis

Total RNA was extracted using HP Total RNA Kit (VWR Scientific, West Chester, PA) according to the manufacturer’s instructions. 1 µg of RNA was reversed transcribed using Superscript II RT according to the manufacturer protocol (Invitrogen, Carlsbad, CA) and quantitative PCR was performed using SYBR Green dye (Roche Scientific, Basel, Switzerland) and a CFX96 instrument (BioRad, Hercules, CA). Primers sequences (IDT, Coralville, IA) used in this study were as follows: COUP TFII: forward, 5’-GCCATAGTCCTGTTCACCTC-3’; reverse, 5’-GGTACTGGCTCCTAACGTATTC-3’; RARB2: forward, 5’-GTGGAGTTTGCTAAACGTCTG-3’; reverse, 5’-TCATGGTGTCTTGTTCTGGG-3’; NGFI-A: forward, 5’-CAGCACCTTCAACCCTCAG-3’; reverse, 5’- AGTCGAGTGGTTTGGCTG-3’; 18S: forward, 5’-CAGCCACCCGAGATTGAGCA-3’; reverse, 5’-TAGTAGCGACGGGCGGTGTG-3’.

### Statistical analysis

All of the results are representative of at least three independent experiments. Statistical significance of the GI_50_ values between wild type, mutated, and null p53 cell lines was calculated using a two-sided unpaired Wilcoxon Rank Sum test. Statistical significance of gene expression in the qRT-PCR analysis and apoptosis assays was calculated using a two-sided Student t-test.

## Results

### 11a does not have agonistic effects towards PNR in cell-based assays

**Figure 1:**
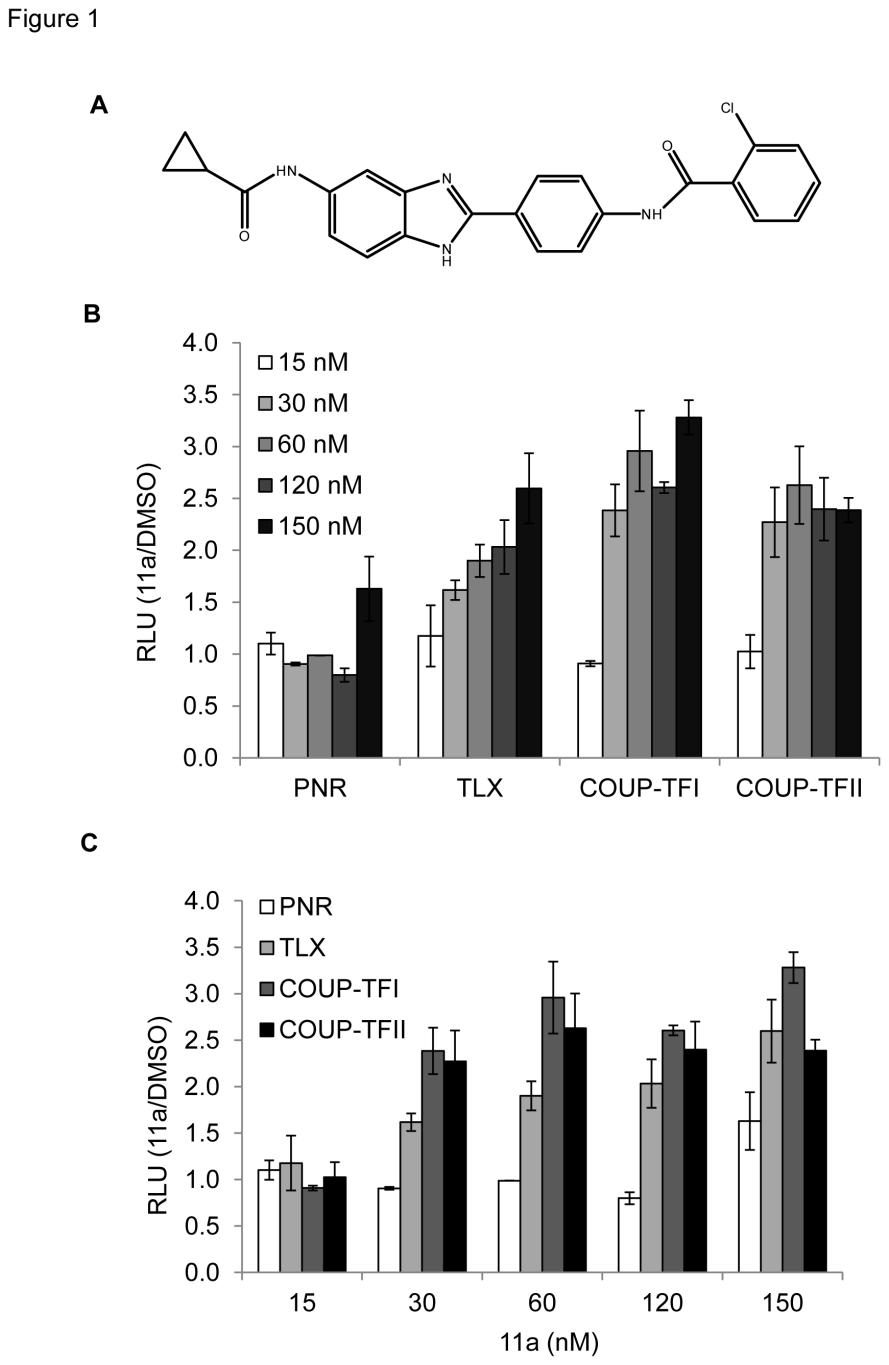
The effect of 11a on PNR, TLX, COUP-TFI and COUP-TFII activation of the DR2-luciferase reporter. (A) Chemical structure of 11a. HEK293T cells transfected with the indicated constructs were treated in triplicate with 0.1% DMSO, 15 nM, 30 nM, 60 nM, 120 nM or 150 nM 11a. Data are expressed as relative luciferase units normalized to the DMSO control ± SD. (B) Comparison between different nuclear receptors with increasing 11a concentrations. (C) Comparison between various doses of 11a with different nuclear receptors.

To investigate cellular functions of PNR, we employed compound 11a (structure shown in Figure 1A), a previously described putative PNR agonist with a cyclopropyl amide group reported to confer a high agonistic activity towards PNR (EC_50_<200 nM) [31]. Compound 11a was synthesized and ^1^H-NMR and mass spectrometry data (Figures S1 and S2) confirmed the correct molecular structure and molecular weight of 11a. TLX, COUP-TFI and COUP-TFII are in the same nuclear receptor subfamily as PNR [36], which bind to a direct repeat of the GGTCA motif with a 2-bp spacing (DR2) [37]. In order to assess the specificity of 11a to PNR, the activation of PNR and these closely related orphan receptors by 11a were compared in a DR2-driven luciferase reporter assay (Figure 1B and 1C). HEK293T cells were transfected with expression vectors for PNR [13], TLX [38], COUP-TFI [39] or COUP-TFII [39] and a DR2-driven luciferase reporter gene, and cells were subsequently treated with 11a using concentrations ranging from 15 nM to 150 nM to minimize the cytotoxic effect. At 15 nM, 11a did not activate any of the nuclear receptors tested. As the concentration increased, 11a slightly activated TLX, COUP-TFI and COUP-TFII in a dose dependent manner. However, PNR activation was seen only at the highest concentration tested (>150 nM) (Figure 1B). We noted that concentrations of 11a greater than 150 nM were cytotoxic and induced severe cell death, which limited the accuracy of luciferase reporter assay. This result indicated that 11a does not have obvious agonistic effects towards PNR. Because PNR was the least activated among the four nuclear receptors tested at the indicated range of 11a concentrations (Figure 1C), our results indicate that the specificity of 11a towards PNR is low and the agonism of 11a is probably not a direct effect, as shown in the NCOR release study where 11a also inhibited TRβ-NCOR and RARα-NCOR interactions [32].

Because 11a activated PNR-related nuclear receptors COUP-TFI and COUP-TFII in the DR2 luciferase assay at the relatively low concentration of 30 nM (Figure 1) and only COUP-TFII could be detected in all breast cancer cell lines [40], we examined whether 11a could alter the expression of COUP-TFII downstream target genes in MCF7 and T47D, two ERα positive breast cancer cell lines. COUP-TFII has been implicated in various cancers for both oncogenic and tumor suppressive effects [41]. In breast cancer cells, RARB2 [42,43] and NGFI-A [44,45] are two well-characterized direct targets up-regulated by COUP-TFII. All-trans retinoic acid (atRA) was previously shown to increase COUP-TFII mRNA level as well as enhancing COUP-TFII downstream target gene expression [46]. Indeed, 1 µM atRA was found to increase COUP-TFII mRNA level by about 1.5- and 2.5-fold in MCF7 and T47D cells, respectively (Figure S3). Interestingly, although 11a did not increase COUP-TFII mRNA levels in the two cell lines, 11a treatment resulted in up-regulation of COUP-TFII target genes. In the MCF7 cell line, 0.1 µM 11a induced NGFI-A gene expression to a similar level as 1 µM atRA. 1 µM 11a induced NGFI-A expression ~5 fold over that of 1 µM atRA (Figure S3A). Because NGFI-A expression is too low to be detected in T47D cells, we measured another COUP-TFII target gene, RARB2. In T47D cells, atRA robustly increased RARB2 mRNA level by 30-fold. Although 11a also increased RARB2 expression in a dose-dependent manner, the magnitude of activation was not comparable to atRA (Figure S3B). These results indicated that 11a possibly regulates COUP-TFII activity in a gene- and cell-specific manner.

**Figure 2:**
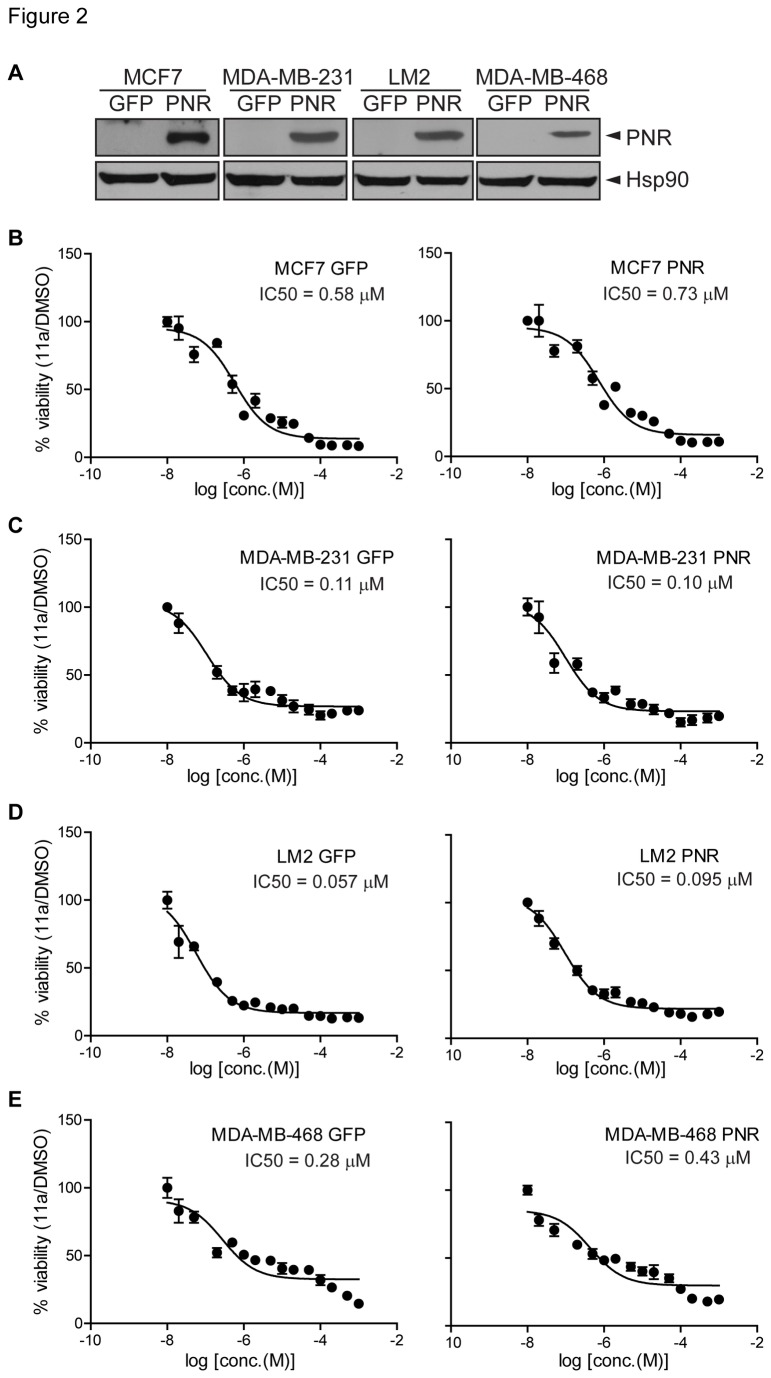
11a cytotoxicity is independent of PNR overexpression in breast cancer cell lines. (A) Breast cancer cells were infected with retroviruses expressing GFP or PNR. PNR expression was detected in the Western blot and Hsp90 was used as the loading control. (B) MCF7, (C) MDA-MB-231, (D) LM2 and (E) MDA-MB-468 breast cancer cells were treated with 11a concentrations ranging from 10^-8^ to 10^-3^ M for 72 hours, and 11a IC_50_ values were obtained by MTT cell proliferation assays.

Since 11a induced cell death in HEK293T cells at higher concentrations and PNR was shown to induce apoptosis in several cell types [28], we further investigated whether 11a-induced cytotoxicity was PNR-mediated. Because PNR is undetectable by western blotting in breast cancer cell lines, several stable PNR overexpression breast cancer cell lines, MCF7, MDA-MB-231, LM2 [34] and MDA-MB-468 cells, were generated (Figure 2A). MTT cell proliferation assays were then used to determine the IC_50_ values for 11a in GFP-expressing control cell lines and PNR-overexpressing cell lines. The IC_50_ values in the cells overexpressing PNR were similar to the corresponding control cell lines (Figure 2B-E), with IC_50_ values ranging from 0.05 to 0.7 µM. Because PNR overexpression did not affect 11a cytotoxicity in any of the cells tested, our results indicate that 11a-induced cytotoxicity is likely independent of PNR in these cells.

### 11a cytotoxicity is correlated with p53 status in NCI-60 cell lines

**Figure 3:**
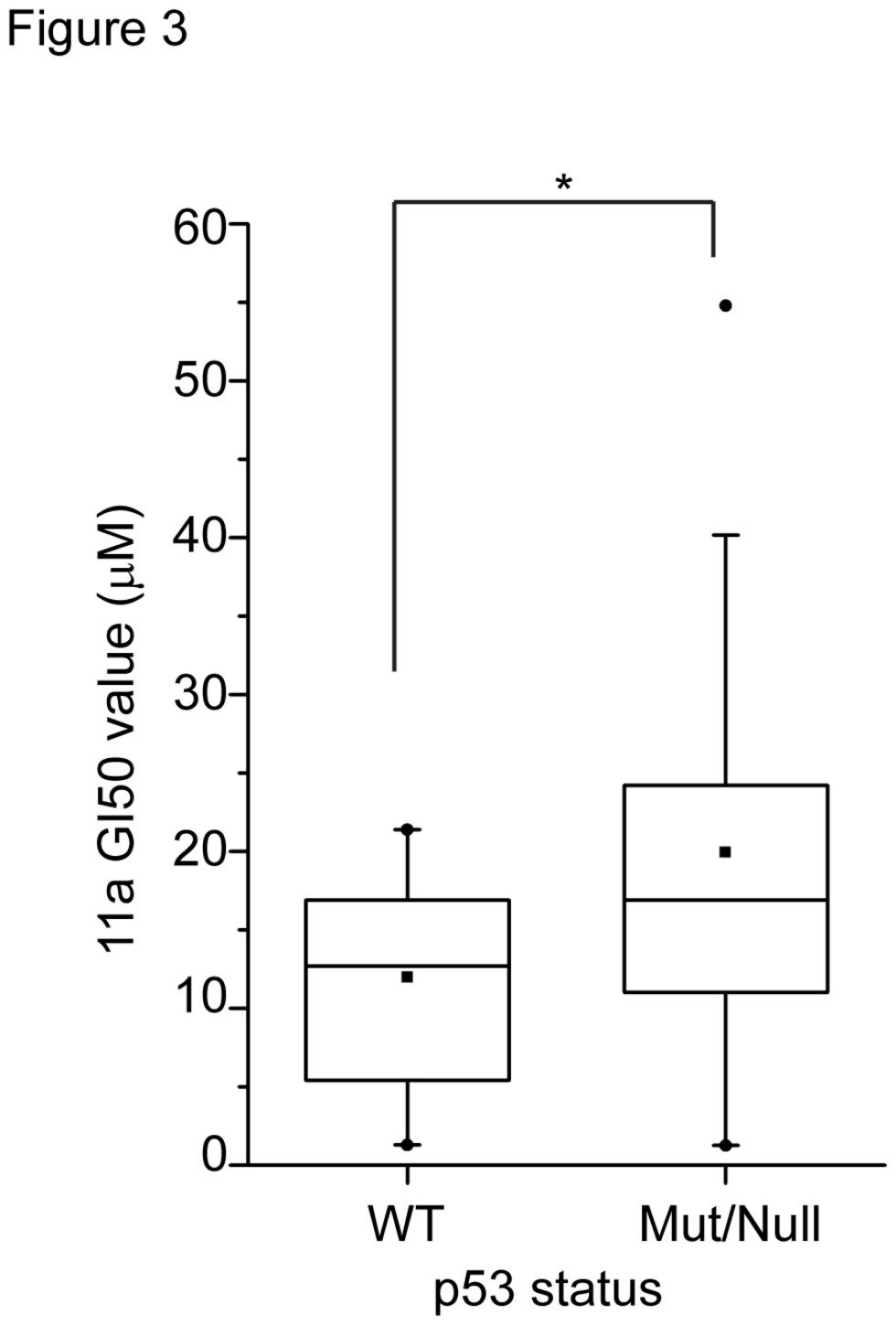
p53 wild type cells exhibit higher sensitivity towards 11a than p53 mutated or null cell lines. 11a GI_50_ values (µM) are plotted against p53 WT and Mut/Null groups in the box chart. Minimum and maximum values, median values and mean values are shown. Significance testing was carried out by two-sided unpaired Wilcoxon rank sum test. *, p<0.05.

To further investigate the mechanism of cytotoxicity and the cellular targets of 11a, we used the Developmental Therapeutics Program (DTP) NCI-60 cell line screening service, a publically accessible service that assists in determining compound cytotoxicity in a panel of 60 cancer cell lines, to assess the cytotoxicity of 11a in 60 cell lines [47]. The 11a cytotoxicity data for 58 of NCI-60 cell lines were received from DTP and GI_50_ data are shown in Figures S4-S6. This study was comprised of 60 cell lines from 9 different cancer types: leukemia, non-small cell lung cancer, colon cancer, CNS cancer, melanoma, ovarian cancer, renal cancer, prostate cancer and breast cancer. The sulphorhodamine-B (SRB) assay was used to obtain the GI_50_ (50% growth inhibition) values of different cancer cell lines. Despite the wide range of cell lines involved, the GI_50_ values of 11a fell in a narrow range (10^-6^ to 10^-5^ M). Since our previous study suggested that PNR stabilizes p53 by post-translational modification in HeLa and HCT116 cell lines [28], we next examined whether 11a sensitivity was correlated with p53 expression level or mutation status. The p53 mutation status of the NCI-60 cell lines was previously determined [48]. The 58 cell lines we received GI_50_ data from DTP can be classified into two categories: p53 wild type and p53 mutated/null (Table 1). By comparing the GI_50_ values of the two groups (Figure 3), we found that p53 wild type cell lines were significantly more sensitive than p53 mutated or null cell lines, with average GI_50_ values 12.0 µM and 19.9 µM respectively (p=0.039, two-sided). These results implicate p53 as a putative determinant of 11a-induced cytotoxicity.

**Table 1:** 11a cytotoxicity results for the 58 cell lines in the NCI60 cell line screening.

p53 WT		p53 Mut/Null					
CELL LINE	conc. (µM)	CELL LINE	conc. (µM)	CELL LINE	conc. (µM)	CELL LINE	conc. (µM)
SR	12.70	HL-60	8.27	KM12	20.90	OVCAR-5	51.50
A549	16.20	K-562	3.59	SW-620	30.50	OVCAR-8	13.30
NCI-H460	18.20	MOLT-4	7.36	SF-268	38.20	ADR-RES	3.16
HCT-116	5.39	RPMI-8226	1.24	SF-295	5.27	SKOV3	40.20
LOX IMVI	7.10	EKVX	2.84	SF-539	24.20	786-0	21.10
MALME-3M	20.90	HOP-62	19.40	SNB-19	32.40	RXF 393	26.30
SK-MEL-5	1.28	HOP-92	17.60	SNB-75	18.50	SN12C	27.50
UACC-257	3.46	NCI-H226	16.90	U251	21.40	TK-10	29.80
UACC-62	21.40	NCI-H23	7.49	M14	16.80	PC-3	12.10
A498	11.60	NCI-H322M	54.80	MDA-MB-435	14.50	DU-145	37.90
ACHN	13.70	NCI-H522	13.60	SK-MEL-2	22.80	MDA-MB-231	16.50
CAKI-1	14.20	COLO 205	12.40	SK-MEL-28	18.50	HS578T	53.40
UO-31	16.90	HCC-2998	22.30	IGROV1	24.20	BT-549	2.00
MCF7	4.35	HCT-15	16.80	OVCAR-3	15.70	T-47D	6.34
average GI50	11.96	HT29	15.90	OVCAR-4	11.00	average GI50	19.92

GI_50_ values and p53 status (WT: wild type; Mut/Null: mutated or null) are shown for each cell line.

### Apoptosis is not the major mechanism accounting for 11a-mediated cytotoxicity

**Figure 4:**
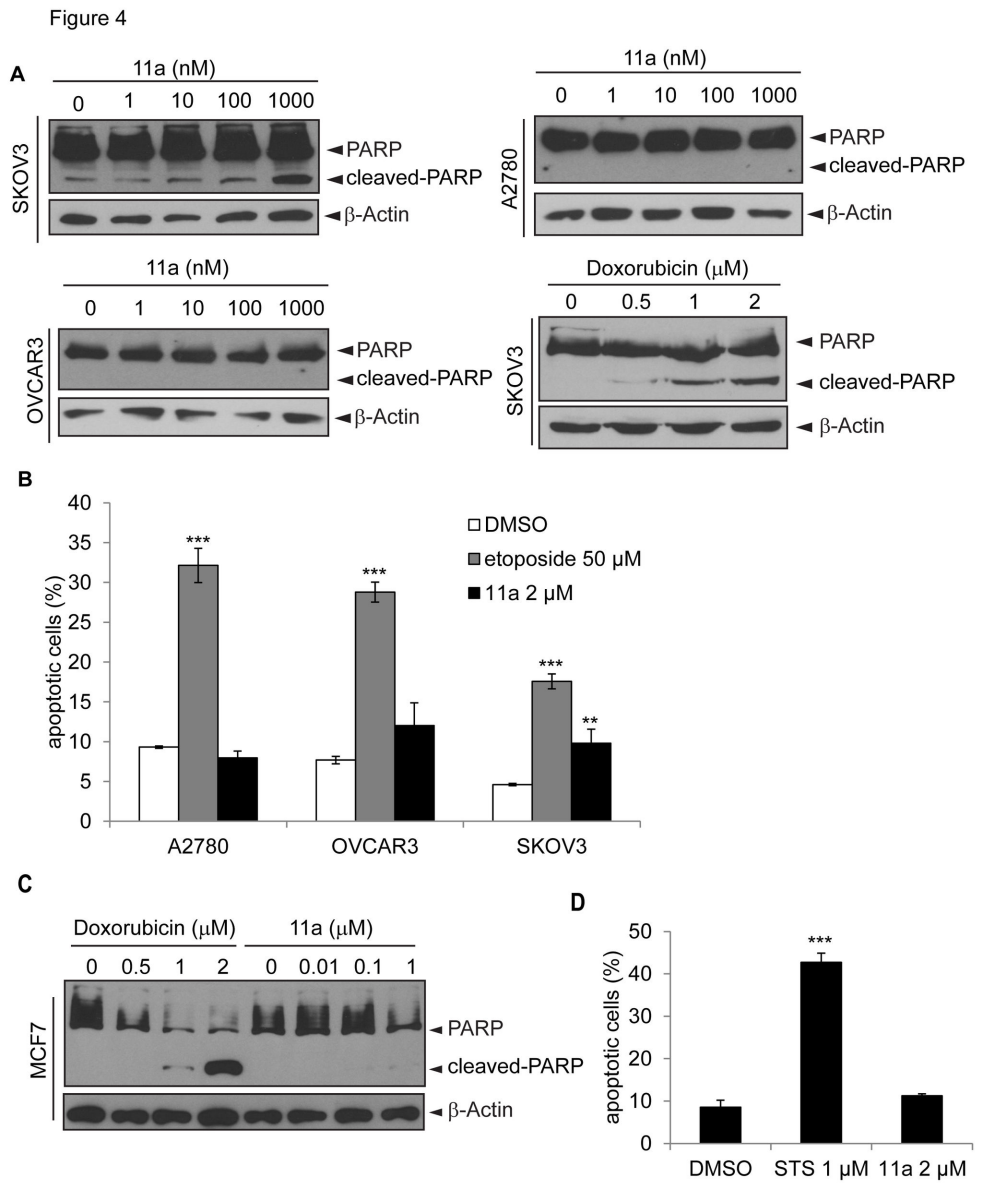
11a induces minimal cell apoptosis in ovarian cancer cell lines and a breast cancer cell line. SKOV3, A2780 and OVCAR3 ovarian cancer cells (A) and MCF7 breast cancer cells (C) were treated with the indicated doses of 11a or doxorubicin for 24 hours. Total cell lysates were probed for PARP cleavage using anti-PARP antibody in western blots. β-Actin was used as a loading control. The black arrows indicate positions of non-cleaved and cleaved PARP proteins. (B) and (D) After 24 hours treatment with 2 µM 11a, cells were collected and stained with Annexin V/PI and subjected to flow cytometry. 50 µM etoposide (B) or 1 µM staurosporine (STS) (D) served as positive controls for apoptosis. The statistical significance were shown as **p<0.01, ***p<0.001 compared with DMSO control.

To study the mechanism of 11a induced cytotoxicity, we selected three ovarian cancer cell lines with representative p53 mutation status: SKOV3 (p53 null), A2780 (p53 wild type) and OVCAR3 (p53 mutation, p.R248Q) [49]. These cells were treated with increasing concentrations of 11a (0 to 1 µM), and the ratio of cleaved PARP to total PARP was used as an indicator of apoptosis [50]. Doxorubicin was used as a positive control to induce apoptosis in SKOV3 cells (Figure 4A). Even at the highest concentrations tested, 11a only modestly induced PARP cleavage in SKOV3 cells but not in A2780 or OVCAR3 cells (Figure 4A). However, the basal level of cleaved PARP was also higher in SKOV3 cells as compared with the other cell lines. To quantitatively investigate the apoptotic effect of 11a, Annexin V/PI double staining was performed. Consistent with the cleaved-PARP assays, 11a only modestly induced apoptosis in SKOV3 cells but not in A2780 or OVCAR3 cells using etoposide as the positive control (Figure 4B and Figure S7). Similar effects were observed in MCF7 breast cancer cell line, where both doxorubicin and staurosporine induced significant apoptosis while 11a did not induce apoptosis at the tested concentrations (Figure 4C and 4D). Collectively, these data indicate that apoptosis is not the main mechanism accounting for 11a-induced cytotoxicity.

### 11a induces G_1_/S cell cycle arrest in a p53-dependent manner

**Figure 5:**
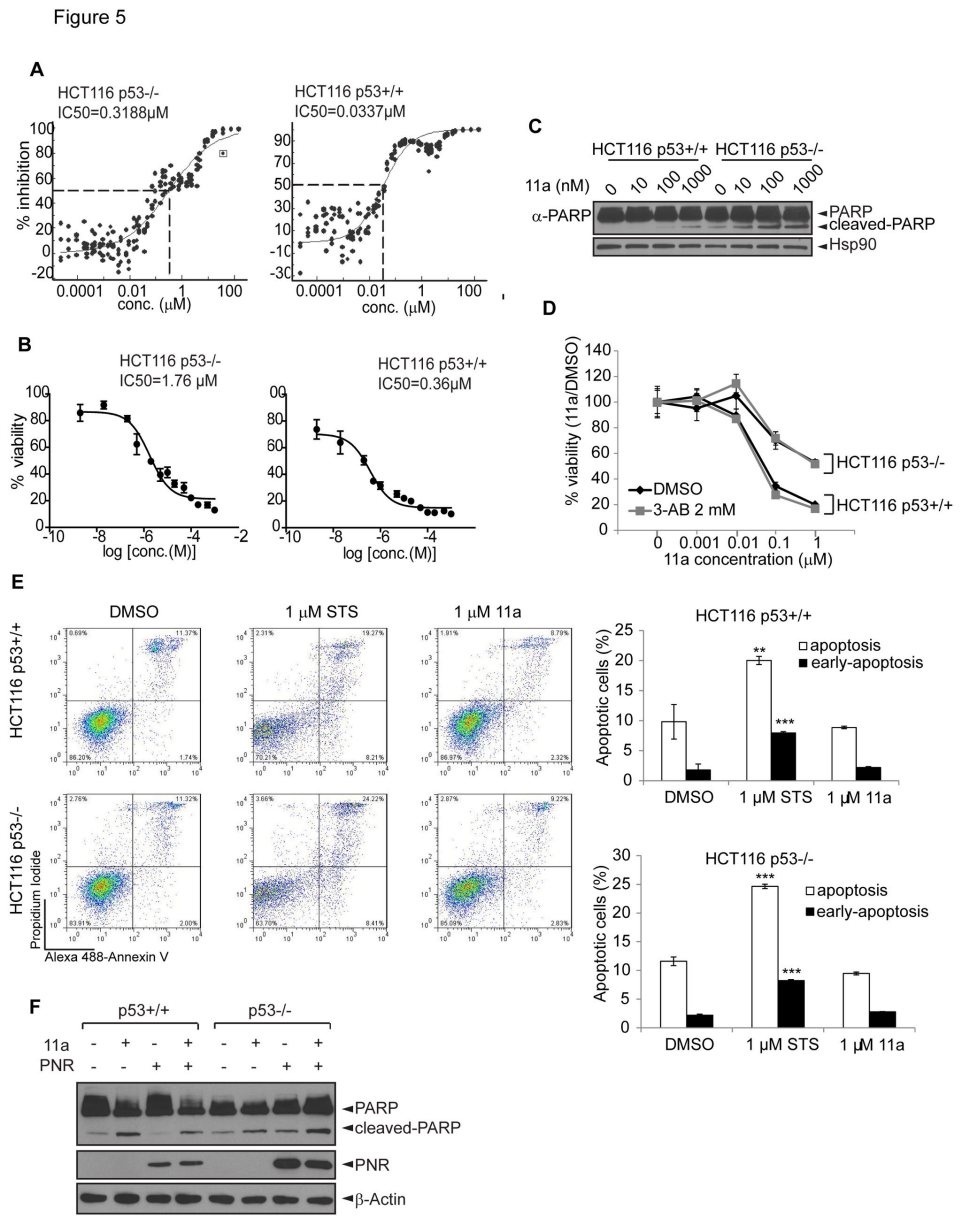
Isogenic HCT116 p53+/+ and p53-/- cell lines show differential sensitivity towards 11a. (A) IC_50_ values of 11a in p53+/+ and p53-/- HCT116 cell lines. The cells were seeded in quadruplicate in the 384-well plates and treated with the indicated concentrations of 11a for 7 days. The growth inhibition was determined by CellTiter Glo luminescent cell viability assay. (B) IC_50_ values of 11a were examined in the MTT cell viability assays after 72 hours incubation with 11a. (C) The two cell lines were treated with 0, 1, 10, 100 or 1000 nM 11a for 24 hours and subjected to Western blot using anti-PARP antibody to detect PARP cleavage. Hsp90 was used as a loading control. (D) The cells were treated with indicated concentrations of 11a in the presence or absence of 2 mM 3-aminobenzamide (3-AB) for 72 hours and then subjected to MTT assays. (E) After 24 hours treatment with 1 µM 11a, cells were collected and stained with Annexin V/PI and subjected to flow cytometry. 1 µM staurosporine (STS) served as a positive control for apoptosis. The statistical significance were shown as **p<0.01, ***p<0.001 compared with DMSO control. (F) The two cells were transfected with 1 µg GFP or PNR for 24 hours followed by treatment with DMSO or 1 µM 11a for another 24 hours. Western blot was performed to examine the PARP cleavage.

Since 11a failed to induce significant apoptosis in any of the cell lines tested, we hypothesized that 11a-induced cytotoxicity may be attributed to cell cycle arrest, which could induce growth inhibition as determined by the sulforhodamine B (SRB) colorimetric assay used for the NCI-60 cell line cytotoxicity screening. To further discern whether 11a cytotoxicity correlated with p53 status, colorectal cancer isogenic HCT116 p53+/+ and HCT116 p53-/- cell lines were used [35]. Interestingly, these isogenic cell lines exhibited differential sensitivity to 11a. The p53 wild type cell line (IC_50_ = 0.0337 µM) was about 10-fold more sensitive than p53 null cell line (IC_50_ = 0.3188 µM) in a pilot screen performed by the Small Molecule Screening and Synthesis Facility (SMSSF) of University of Wisconsin (Figure 5A). The differential sensitivity was later confirmed using the MTT proliferation assay where p53 wild type cells (IC_50_ = 0.36 µM) were more sensitive than p53 null cells (IC_50_ = 1.76 µM) (Figure 5B). To further investigate the mechanism by which the two isogenic cell lines showed differential sensitivities to 11a, we assessed the apoptotic effects of 11a in these two cell lines. The cells were treated with increasing concentrations of 11a for 24 hours, and apoptosis was measured using a PARP-cleavage assay, in which the PARP cleavage ratio indicates the apoptotic status. Figure 5C shows that only modest PARP cleavage was observed in either p53+/+ or p53-/- HCT116 cells. To examine whether PARP played a role in 11a mediated cytotoxicity, we co-treated cells with 11a and 3-aminobenzamide (3-AB), a specific PARP inhibitor [51]. PARP inhibition did not affect the cytotoxicity of 11a (Figure 5D), indicating that 11a mediated cytotoxicity was independent of PARP activity. The apoptotic effect of 11a was further examined by Annexin V/PI staining. While staurosporine caused severe apoptosis, 11a did not induce any apoptosis in the isogenic cell lines as compared with DMSO control (Figure 5E). Because PNR protein was undetectable in these cells, we over-expressed PNR followed by treatment with 11a. Our results reinforced that the apoptotic effect was independent of PNR (Figure 5F).

**Figure 6:**
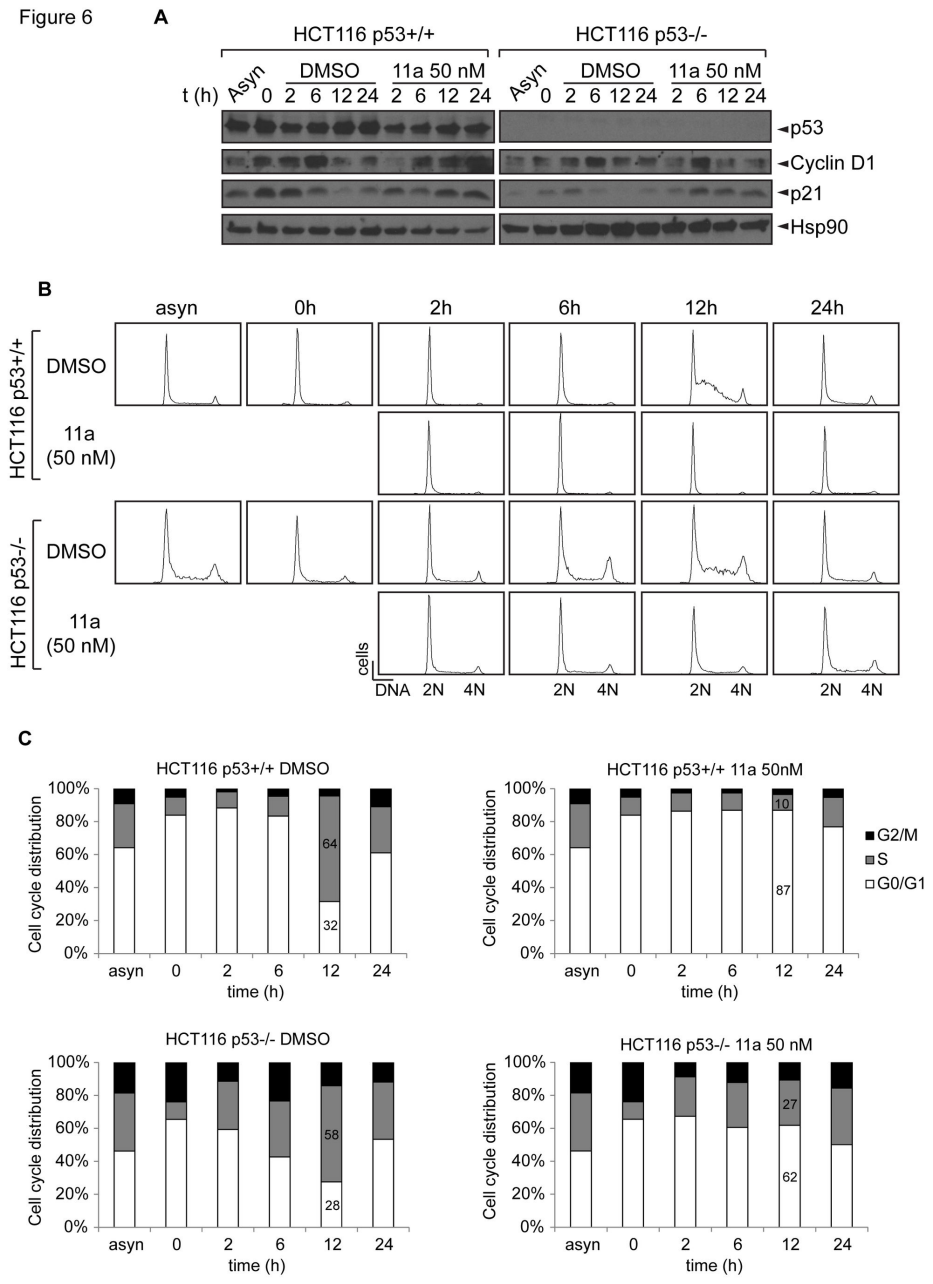
11a induces G_1_/S phase cell cycle arrest in a time dependent manner in HCT116 isogenic cell lines. (A) Western blot against p53, Cyclin D1, p21 and Hsp90, which was used as the loading control (B) Histograms of the FACS analysis (C) Quantification of the FACS analysis and cell cycle distribution of the two cell lines treated with 0.1% DMSO or 50 nM 11a.

**Figure 7:**
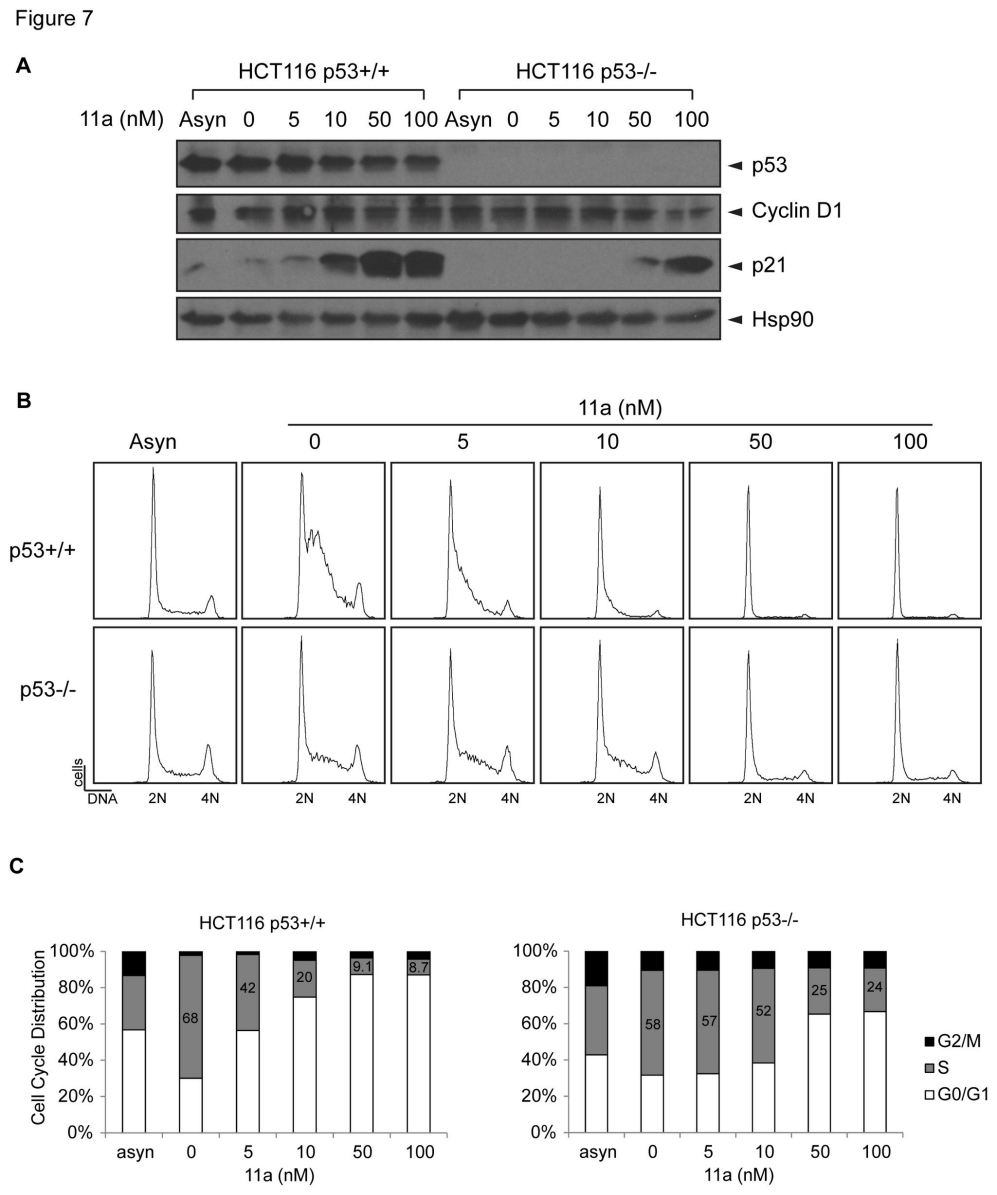
11a induces G_1_/S phase cell cycle arrest in a dose dependent manner in HCT116 isogenic cell lines. (A) Western blot against p53, Cyclin D1, p21 and Hsp90 as the loading control. (B) Histograms of the DNA FACS analysis of the cultures from panel A. (C) Quantification of the FACS analysis and cell cycle distribution of the two cell lines treated with 0, 5, 10, 50, or 100 nM 11a in 0.1% DMSO for 12 hours.

These studies indicate that 11a might have more profound effects on cell cycle arrest than apoptosis. To examine whether 11a induced cell cycle arrest, the cells were synchronized at the G_0_/G_1_ phase by serum starvation for 24 hours. Figure 6 shows the results of the cell cycle profile analysis of synchronous HCT116 cells treated with DMSO or 50 nM 11a immediately after release of serum starvation. When cells were treated with DMSO, the majority of the cells were in S phase after 12 hours (64% for p53+/+ cells and 58% for p53-/- cells), and the cells returned to G_1_ phase 24 hours later. This result is in keeping with the normal cell cycle of 24 hours for these isogenic cell lines. However, when the synchronized p53 wild type cells were treated with 50 nM 11a, a concentration close to the cytotoxicity IC_50_ of 33.7 nM, a G_1_/S phase cell cycle arrest occurred for up to 24 hours (Figure 6B). After treatment with 11a for 12 hours, only 10% of the cells returned to S phase compared with 64% treated with DMSO, and the majority of the 11a-treated cells were arrested at G_0_/G_1_ phase (87%). The G_1_/S phase cell cycle arrest was retained after 24 hours (Figure 6B). The 11a-treated p53 null cells also experienced a G_1_/S phase arrest after 12 hours (27% S phase population with 11a treatment compared with 58% with DMSO treatment); however, the checkpoint was recovered after 24 hours (Figure 6B), indicating that the cell cycle arrest was not as severe as that of the p53 wild type HCT116 cells. The protein levels of p21 and cyclin D1 oscillate during the cell cycle and are involved in G_1_-S phase transition. Cyclin D1 increases during G_1_ phase when it forms a complex with CDK4/6 mediating the phosphorylation of pRb to facilitate G_1_-S phase transition [52,53]. Cyclin D1 level remains low during S phase. p21, the cyclin dependent kinase inhibitor, which hinders the kinase activity of CDK2-cyclin E, also has higher level in G_1_ phase but decreases when the cells enter S phase [54]. We used these two cell cycle biomarkers to monitor the cell cycle progression with DMSO or 11a treatment, and the oscillation of the level of p21 and cyclin D1 indicated the corresponding cell cycle phases (Figure 6A and Figure 7A). The persistently high p21 and cyclin D1 protein levels in HCT116 p53+/+ cells after 11a treatment compared with HCT116 p53-/- cells also supported the G_1_/S phase cell cycle arrest. An assessment of the cell cycle distribution in response to increasing doses of 11a (Figure 7B,C) further revealed that p53+/+ cells were more sensitive to 11a with regards to induction of the G_1_/S arrest as compared with p53-/- cells. For example, 5 nM 11a induced cell cycle arrest in p53+/+ cells to a similar degree as 50 nM 11a treatment in p53-/- cells, suggesting that p53+/+ cells were more vulnerable to 11a induced G1/S arrest than p53-/- cells.

## Discussion

In this study, we evaluated the dependency of 11a cytotoxicity on PNR expression and further investigated the mechanism of 11a cellular cytotoxicity using various cell-based assays. Although 11a was originally reported as a synthetic agonist for PNR, it was found not to be a direct PNR agonist in the recently developed TR-FRET assay which measured PNR-RetCoR dissociation [32]. Consistent with this finding, our cell-based assays similarly showed that 11a was unlikely to be a direct agonist for PNR. We employed luciferase reporter assays to compare the ability of 11a to activate different subfamily II nuclear receptors, of which PNR is a member [36]. However, our results showed that 11a is least potent in PNR activation, whereas 11a could weakly activate other members of subfamily II such as TLX, COUP-TFI and COUP-TFII at the different concentrations tested. Further studies using stable cell lines overexpressing PNR confirmed that the 11a-induced cytotoxic effects are PNR independent. Together, our results indicate that 11a may have cellular targets other than PNR to confer cytotoxic effects.

Although the cellular targets of 11a remain to be determined, 11a was found to activate COUP-TFII in DR2-luciferase reporter assay and COUP-TFII target genes in two breast cancer cell lines. The only other known weak agonist for COUP-TFII is atRA [44]. atRA was shown to activate COUP-TFII on the NGFI-A promoter in the luciferase reporter assay [44]. Induction of RARB2 by atRA causes growth inhibition and apoptosis in cancer cells and this process requires the orphan nuclear receptor COUP-TFII [42]. Since 11a activated COUP-TFII in the DR2 luciferase assay (Figure 1B) and induced RARB2 and NGFI-A gene expression to a comparable level as atRA (Figure S3), it is possible that 11a could serve as an agonist for COUP-TFII and substitute atRA in some cancer treatment. For example, all-trans retinoic acid has long been used for the treatment of acute promyelocytic leukemia (APL) and were shown to inhibit solid tumor growth [55], however, the strong cytotoxicity prevents its wide use in cancer treatment. The concentrations of RAs required for activation of COUP-TFII are 10-100 times higher than the physiological levels [44], on the contrary, 11a at 10-time lower concentration could manifest the same effect in the COUP-TFII target gene activation (Figure 3). Whether 11a-induced cytotoxicity is at least in part mediated through COUP-TFII is worth further investigation. Future works are warranted to determine whether 11a directly binds COUP-TFII and other NRs, and whether 11a-induced gene expression change and cytotoxicity effects are dependent on COUP-TFII by knocking down COUP-TFII in breast cancer cell lines.

Even though PNR is not the cellular target of 11a, 11a might still have cytotoxic effects and thus can be explored as an anti-cancer drug. To test this possibility, we performed systematic cytotoxicity studies using the NCI-60 cell lines. Our results revealed that 11a could induce cytotoxicity in a broad range of cell lines, which is contradictory to a previous report showing that 11a was non-toxic in CHO cells [31]. The cytotoxicity of 11a was evaluated in a NCI-60 cell line screen using the SRB cell viability assay, with the advantage of differentiating cell killing (LC_50_, 50% reduction in the measured protein at the end of the drug treatment as compared to that at the beginning) from growth inhibition (GI_50_, 50% reduction in the net protein increase in control cells during the drug incubation) [47,56-58]. The NCI-60 cell line study screens classical and newly synthetic compounds with unique structures and functional groups to assess the mechanism of cytotoxicity and characterize selected cytotoxic effects towards certain cancer types [47], with the aim of finding novel drugs for cancer research and treatment. The sensitivity to 11a is strongly correlated with p53 status, not only in the NCI-60 screen (Figure 3) but also with the HCT116 isogenic cell lines (Figure 5). Because 11a did not strongly induce apoptosis in various cell lines, we focused on G_1_/S cell cycle arrest in isogenic HCT116 cell lines with null or wild type p53. Although p53+/+ and p53-/- HCT116 cell lines both underwent G_1_/S phase cell cycle arrest, the p53 wild type cells exhibited higher sensitivity towards 11a as compared with the p53-/- cells, and the checkpoint could not be recovered even after 24 hours treatment. We concluded that the differential sensitivity was at least partially due to p53 function in HCT116 cell lines. The cell cycle arrest in the p53 null cell line may have been caused by p21 induction (Figures 6A, 7A) and the activity of other players like Cdk2 and Rb, which regulate the G_1_/S phase transition [59-61].

PNR was proposed as a putative therapeutic target for various diseases including p53-positive and ERα-positive cancers. Because endogenous ligands have not been identified for PNR, efforts have been made to identify synthetic PNR agonists. The lack of highly sensitive assays and a crystal structure of PNR greatly limit the discovery of synthetic PNR agonists. 13-cis-retinoic acid, the natural retinal pigment, could only confer agonistic activity towards PNR at non-physiological concentrations [31]. Thus far, only 11a was described as a potent PNR agonist by one study [31], yet this is challenged by a recent study [32]. Our results agree with the latter study, demonstrating that 11a cytotoxicity is independent of PNR. Rather, we show that 11a exhibits differential cytotoxicity in various cancer cell lines, and this cytotoxicity correlates with p53 mutation status. It appears that 11a could have multiple cellular targets, as do some other cancer targeting drugs. To shed insights into the cellular targets of 11a, we extracted TGI (total growth inhibition) and LC_50_ (50% lethal concentration) data from the NCI-60 cell line screen database to identify compounds with similar cell killing profiles as 11a. A strong correlation (>0.8) was found between 11a and the compounds morpholino-ADR, didemnin B, vincristine sulfate, and tetraplatin, which are well-known anti-cancer drugs used for cancer treatment. This correlation may indicate a potentially similar drug action in cancer cells and the utility of 11a to antagonize tumor progression. More pharmacokinetic studies need to be performed to characterize 11a, and more importantly, real PNR agonists remain to be identified to assess the utility of PNR as a therapeutic target for retinal diseases and cancers.

## Supporting Information

Figure S1 **Molecular weight of 11a determined by time-of-flight mass spectrometry.** (A) The ESI-EMM-TOF spectrum with m/z range from 0 to 1400. (B) Zoom-in of the ESI-EMM-TOF spectrum with m/z range from 400 to 560. (C) Calculated m/z for C_24_H_19_ClN_4_O_2_ is 431.1270, with Δ<1 ppm from the obtained m/z of 431.1267.
(TIF)

Figure S2 **Molecular structure of 11a determined by ^1^H NMR.** The structure of the synthesized compound 11a was determined with ^1^H NMR by the Small Molecule Screening Facility of UW-Madison. The experimentally determined mass is 430.12, which is almost identical to the expected molecular weight of 430.89.
(TIF)

Figure S3 **11a induction of RARB2 and NGFI-A gene expression.** (A) MCF7 (B) T47D breast cancer cells were treated with DMSO, 1 µM atRA, 0.1 µM 11a or 1 µM 11a for 24 hours prior to RNA extraction and reverse transcription. COUP-TFII, RARB2 and NGFI-A gene expression was examined by qRT-PCR. The error bars represent ± SD values. The significance of gene expression up-regulation were shown as *p<0.05, **p<0.01, ***p<0.001 compared to DMSO control.
(TIF)

Figure S4 **Dose response curves of NCI-60 cell lines.** The dose response curves are plotted individually for the nine cancer types. Percentage growth was shown as a function of five concentrations ranging from 10^-8^ to 10^-4^ molar of 11a.
(TIF)

Figure S5 **GI_50_, TGI and LC_50_ values of 11a of the NCI-60 cell lines.** The in vitro testing results show the mean optical densities and percent growth with each dose of 11a, and the 50% of growth inhibition (GI_50_), total growth inhibition (TGI) and 50% lethal concentration (LC_50_) values for each cell line.
(TIF)

Figure S6 **Mean graphs of 11a NCI-60 cell line screening.** The GI_50_, TGI and LC_50_ values are plotted in the mean graphs. The mean of Log_50_ values of the 60 cell lines is set as 0 for all the three parameters.
(TIF)

Figure S7 **Apoptotic effects of 11a on ovarian and breast cancer cell lines.** After 24 hours treatment with 2 µM 11a, (A) A2780 (B) OVCAR3 (C) SKOV3 and (D) MCF7 cells were collected and stained with Annexin V/PI and subjected to flow cytometry. 50 µM etoposide or 1 µM staurosporine (STS) served as positive controls for apoptosis. The early and late apoptosis were determined with FlowJo analysis. Representative stainings were shown.
(TIF)
