# Supplementary figures and images for: Systematic Analyses of the Cytotoxic Effects of Compound 11a, a Putative Synthetic Agonist of Photoreceptor-Specific Nuclear Receptor (PNR), in Cancer Cell Lines
Source: PLoS One. 2013 Sep 16;8(9):e75198. doi: 10.1371/journal.pone.0075198 (PMC3774666; doi:10.1371/journal.pone.0075198)

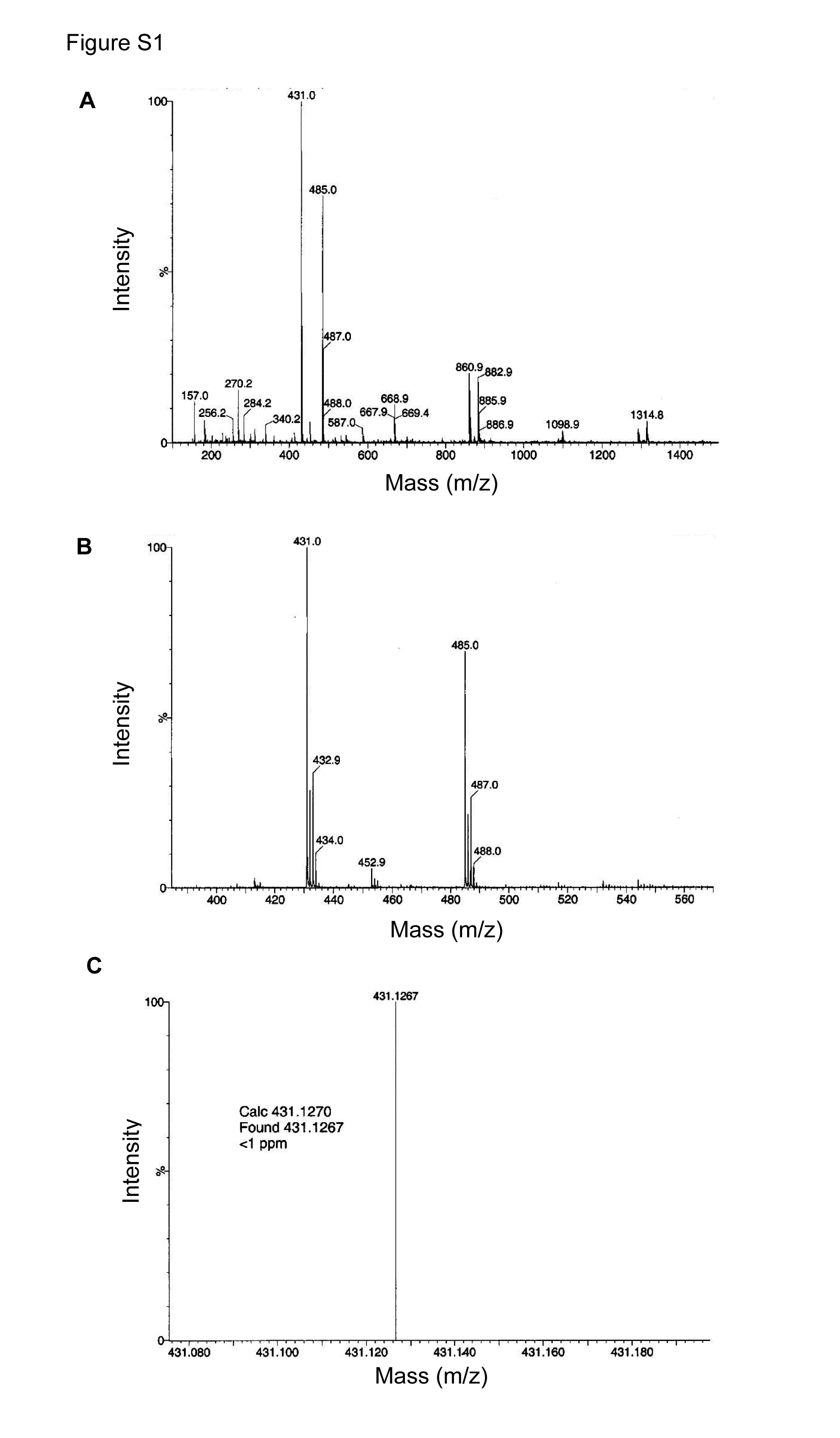

Supplement: Figure S1 — Molecular weight of 11a determined by time-of-flight mass spectrometry. (A) The ESI-EMM-TOF spectrum with m/z range from 0 to 1400. (B) Zoom-in of the ESI-EMM-TOF spectrum with m/z range from 400 to 560. (C) Calculated m/z for C24H19ClN4O2 is 431.1270, with Δ<1 ppm from the obtained m/z of 431.1267. (TIF) [file pone.0075198.s001.tif]

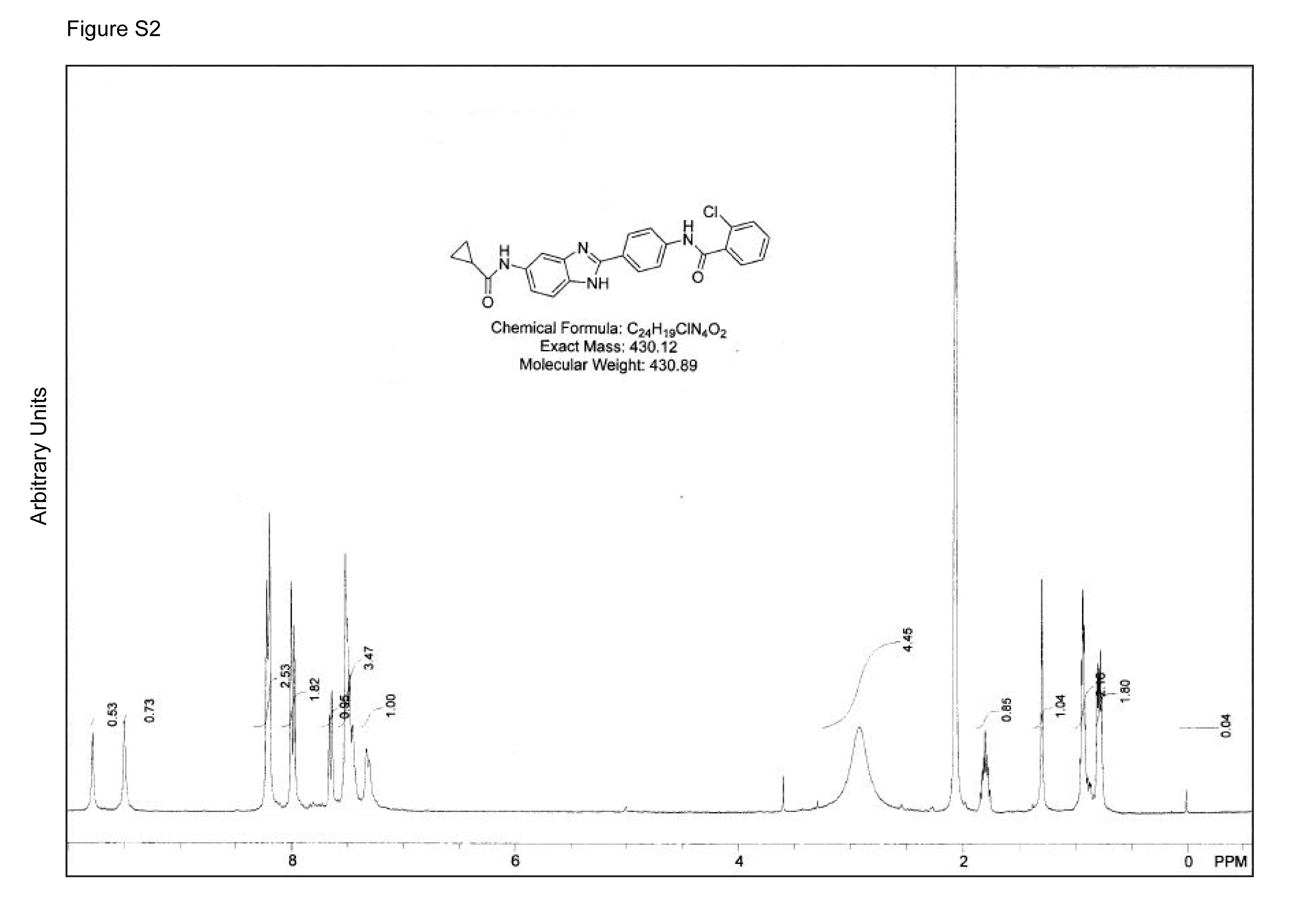

Supplement: Figure S2 — Molecular structure of 11a determined by 1H NMR. The structure of the synthesized compound 11a was determined with 1H NMR by the Small Molecule Screening Facility of UW-Madison. The experimentally determined mass is 430.12, which is almost identical to the expected molecular weight of 430.89. (TIF) [file pone.0075198.s002.tif]

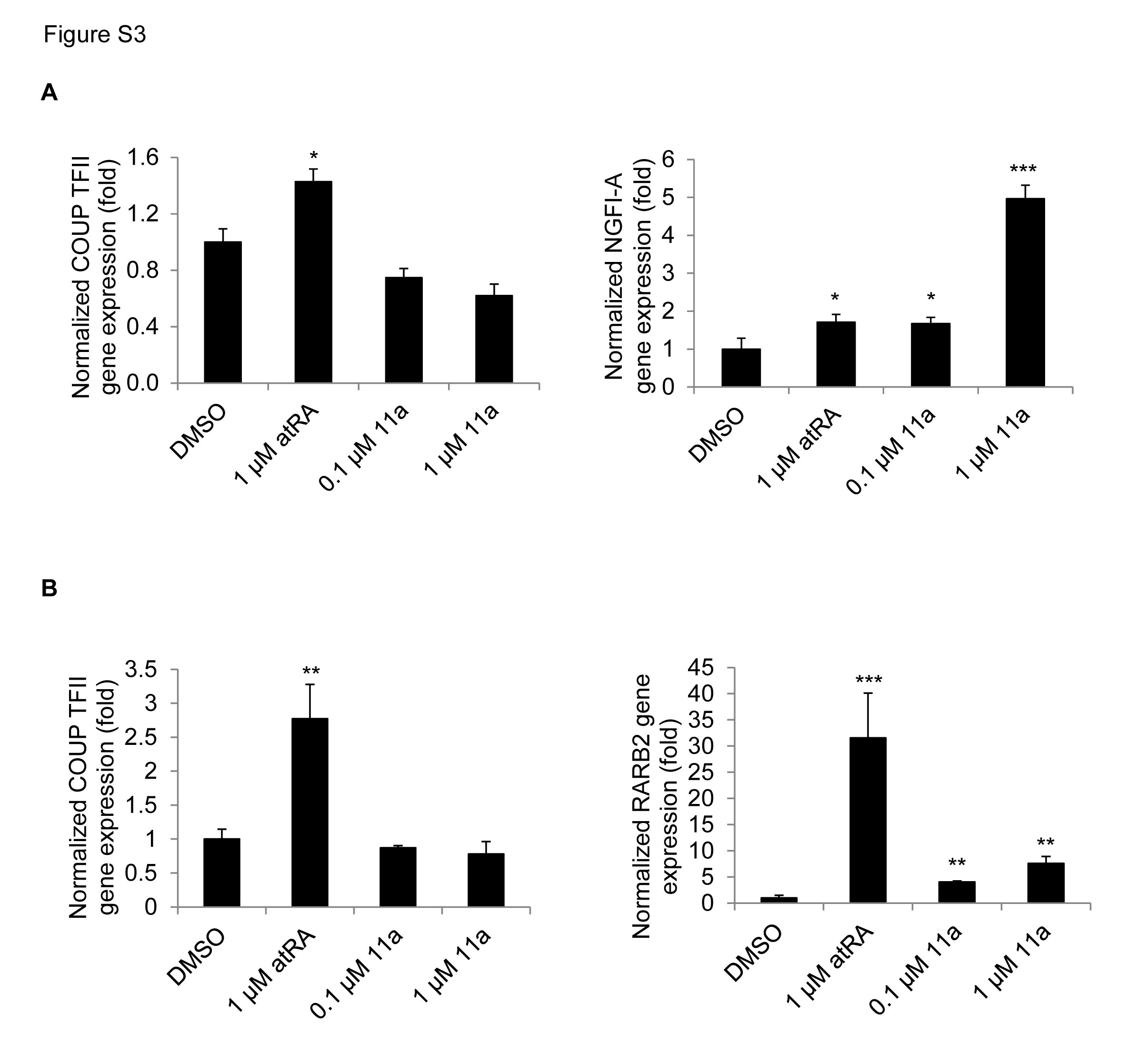

Supplement: Figure S3 — 11a induction of RARB2 and NGFI-A gene expression. (A) MCF7 (B) T47D breast cancer cells were treated with DMSO, 1 µM atRA, 0.1 µM 11a or 1 µM 11a for 24 hours prior to RNA extraction and reverse transcription. COUP-TFII, RARB2 and NGFI-A gene expression was examined by qRT-PCR. The error bars represent ± SD values. The significance of gene expression up-regulation were shown as *p<0.05, **p<0.01, ***p<0.001 compared to DMSO control. (TIF) [file pone.0075198.s003.tif]

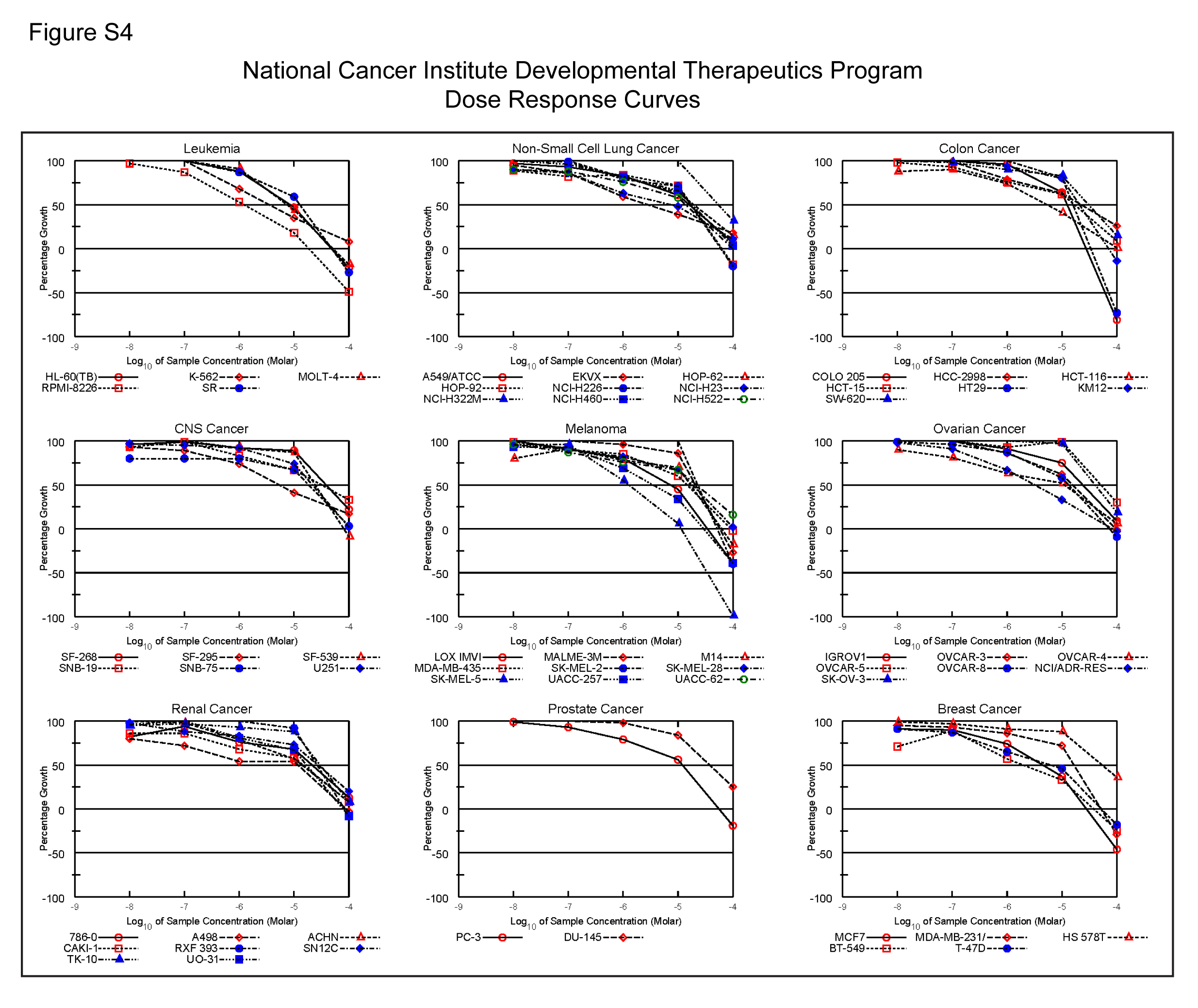

Supplement: Figure S4 — Dose response curves of NCI-60 cell lines. The dose response curves are plotted individually for the nine cancer types. Percentage growth was shown as a function of five concentrations ranging from 10-8 to 10-4 molar of 11a. (TIF) [file pone.0075198.s004.tif]

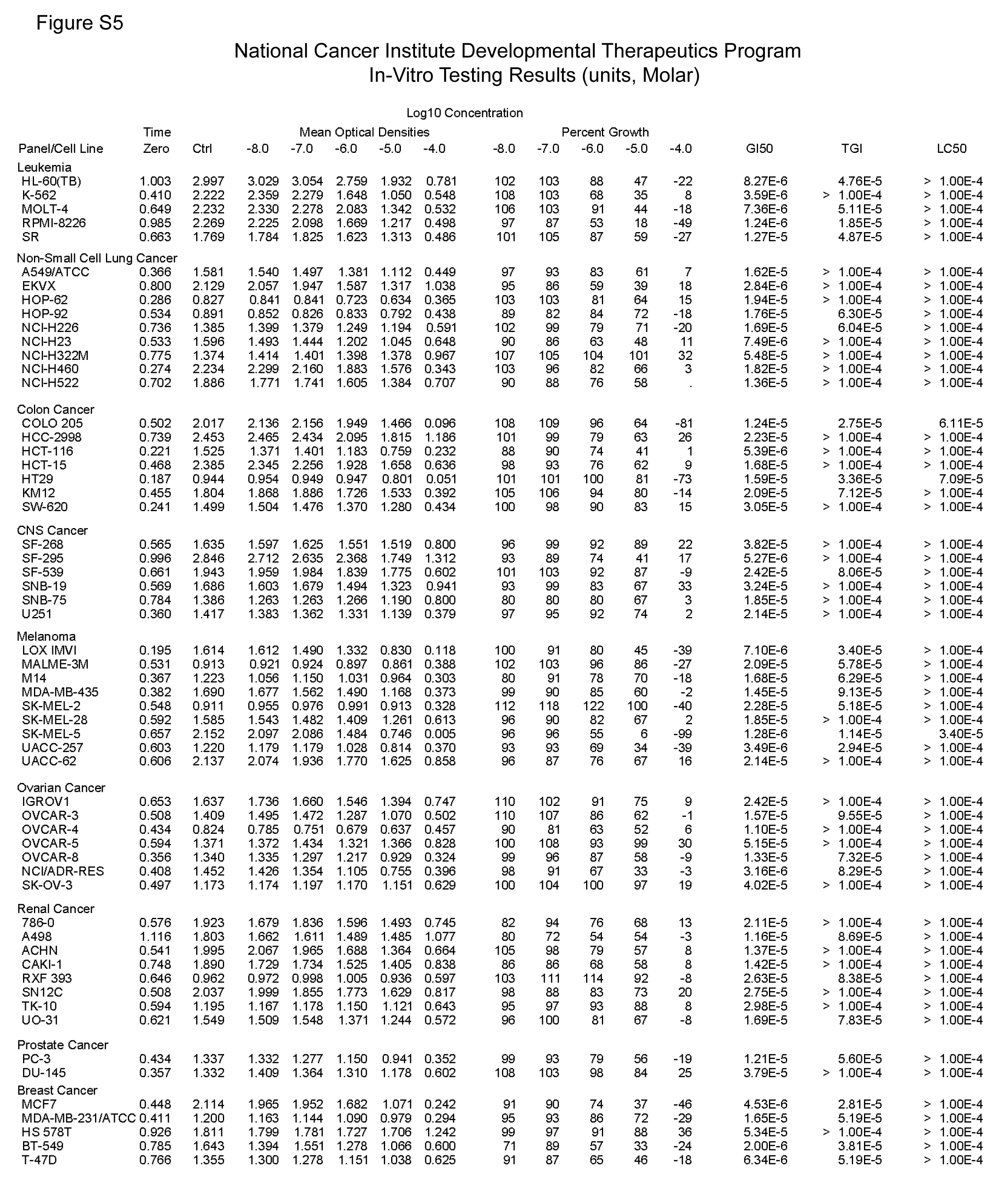

Supplement: Figure S5 — GI50, TGI and LC50 values of 11a of the NCI-60 cell lines. The in vitro testing results show the mean optical densities and percent growth with each dose of 11a, and the 50% of growth inhibition (GI50), total growth inhibition (TGI) and 50% lethal concentration (LC50) values for each cell line. (TIF) [file pone.0075198.s005.tif]

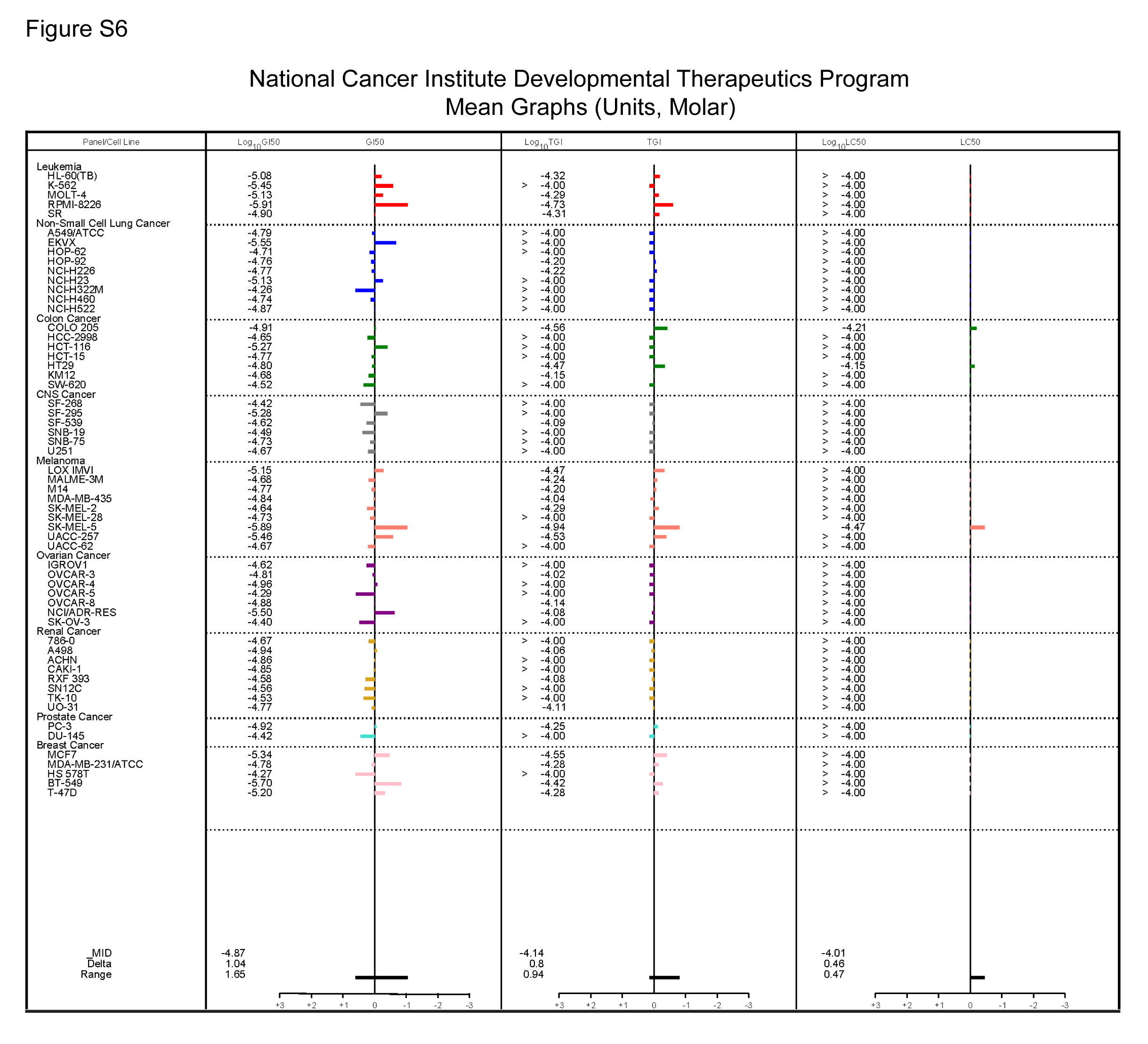

Supplement: Figure S6 — Mean graphs of 11a NCI-60 cell line screening. The GI50, TGI and LC50 values are plotted in the mean graphs. The mean of Log50 values of the 60 cell lines is set as 0 for all the three parameters. (TIF) [file pone.0075198.s006.tif]

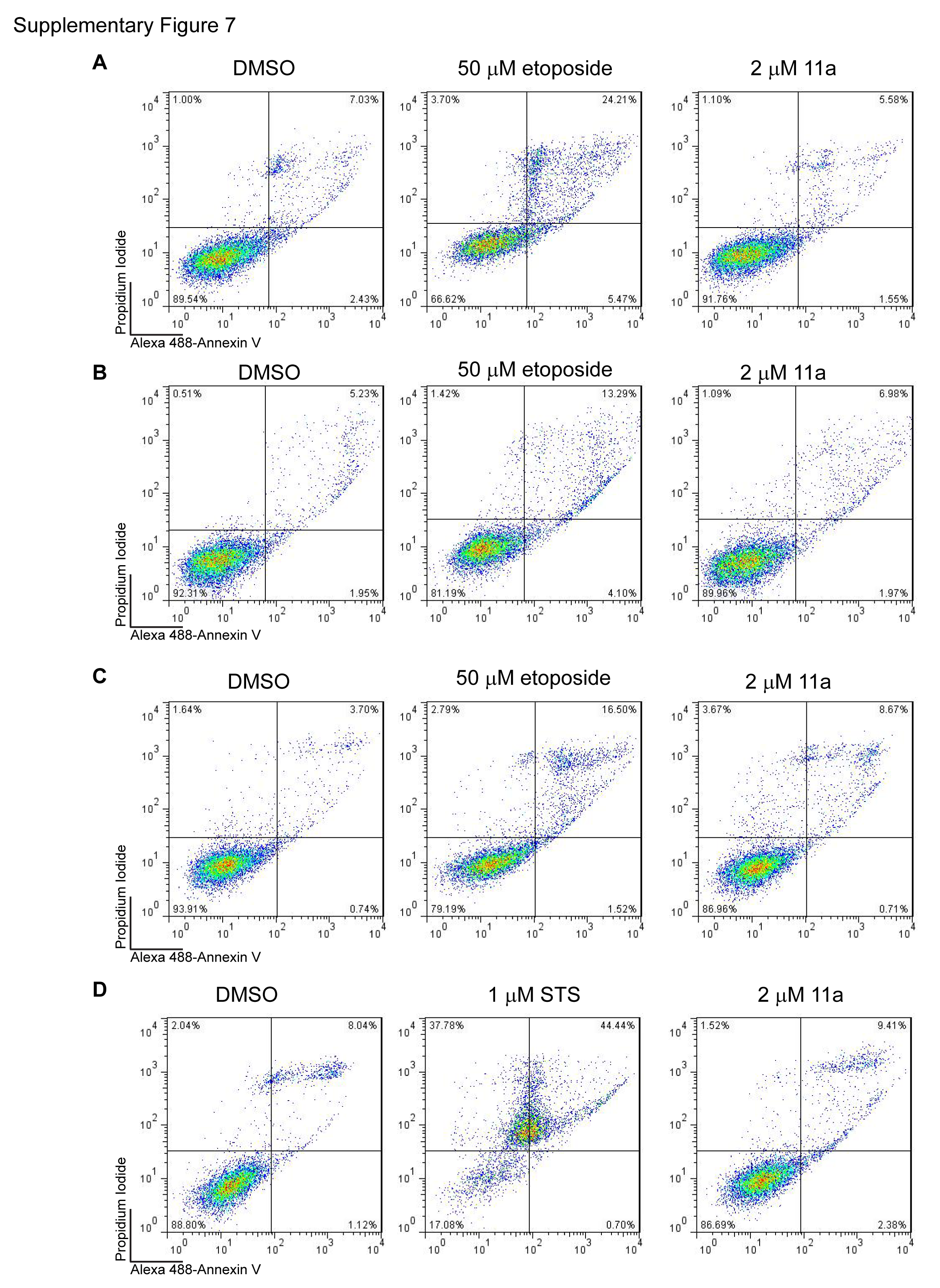

Supplement: Figure S7 — Apoptotic effects of 11a on ovarian and breast cancer cell lines. After 24 hours treatment with 2 µM 11a, (A) A2780 (B) OVCAR3 (C) SKOV3 and (D) MCF7 cells were collected and stained with Annexin V/PI and subjected to flow cytometry. 50 µM etoposide or 1 µM staurosporine (STS) served as positive controls for apoptosis. The early and late apoptosis were determined with FlowJo analysis. Representative stainings were shown. (TIF) [file pone.0075198.s007.tif]
